# Effects of dietary and health factors on nutritional status of children in pastoral settings in Borana, southern Ethiopia, August–October 2015

**DOI:** 10.1186/s13690-021-00692-3

**Published:** 2021-09-22

**Authors:** Bekele Megersa, Abebe Haile, Uriel Kitron

**Affiliations:** 1 College of Veterinary Medicine and Agriculture, Addis Ababa University, Bishoftu, Ethiopia; 2 Center for Food Security Studies, College of Development Studies, Addis Ababa University, Addis Ababa, Ethiopia; 3 Department of Environmental Sciences, Emory University, 30322 Atlanta, GA USA

**Keywords:** Anthropometry, Children, Determinants, Malnutrition, Pastoral system, Borana

## Abstract

**Background:**

Childhood undernourishment is a major public health problem globally, and being responsible for higher mortalities in children and enormous health costs in sub-Saharan Africa. However, scarcity of data on the magnitude of malnutrition and its underlying causes, especially in the pastoral system, limits the effectiveness of potential interventions. This study addresses the nutritional status and factors associated with malnutrition among children in Borana pastoral system, southern Ethiopia.

**Methods:**

A community based cross-sectional study, using multistage cluster sampling, was conducted from August to October 2015. Dietary diversity score (DDS), milk and meal frequencies, anthropometric measurements, and socio-economic variables were recorded for 538 children aged 6–59 months. Multivariable generalized linear model (GLM) with log link function was applied to ascertain determinants of malnutrition. The strength of association was assessed based on prevalence ratio (PR).

**Results:**

Prevalence of underweight, stunting, and wasting were 28.3 % (95 % CI: 24.4–32.1), 41.1 % (95 % CI: 36.7–45.1), and 9.8 % (95 % CI: 7.3, 12.4), respectively. Children who consumed more diverse foods were at a lower risk of being underweight (PR = 0.72, 95 % CL: 0.59–0.88), stunted (PR = 0.80, 95 % CL: 0.68–0.93) and wasted (PR = 0.42, 95 % CL: 0.27–0.66). Intake of increased milk frequency was also associated with lower risk of underweight (PR = 0.86, 95 %CL: 0.76–0.97), stunting (PR = 0.83, 95 %CL: 0.75–0.91) and wasting (PR = 0.73, 95 %CL: 0.56–0.96). The risk of underweight (PR = 1.02, 95 %CL: 1.01–1.03), stunting (PR = 1.01, 95 %CL: 1.00–1.02) and wasting (PR = 1.01, 95 %CL: 1.00–1.04) had increased with age, and no difference was observed between boys and girls. Children who lived far away from health care facilities were 1.2 and 2.4 times more likely to be stunted and wasted, respectively than those residing near a health care facility. Ownership of toilet and living close to market were associated with reduced stunting, whereas illness was associated with increased risk of underweight.

**Conclusions:**

The high prevalence of stunting among pastoral children is a serious public health concern and calls for urgent action. Association of nutritional status of children with dietary intake, and health status, access to health services and toilet availability underlines the need for improved nutrition practices, health care facilities and sanitary conditions in the study area.

## Background

Adequate nutrition is vitally important for the healthy growth, as well as for optimal physical and mental development of children. Undernutrition, particularly during the critical periods for proper brain development and linear growth, prevents children from attaining their full physical and mental potential, and compromises their health status and productive potentials [1]. Consequently, a high prevalence of malnourished adults in a population hampers the entire economic and social development of a nation, and further results in a vicious cycle of poverty [2]. Malnutrition is a major public health problem worldwide that contributes to high morbidity and mortality rates, and is responsible for 45 % of all deaths in children < 5 years of age [3].

In recent years, the proportion of malnourished children has shown a decreasing trend globally with 24.5 % stunted, 15 % underweight and 7.7 % wasted children in 2015 [4]. However, prevalence of malnutrition and its impacts remains highest in Sub-Saharan Africa (SSA) with 39 %, 25% and 10 % of the children reported to be stunted, underweight and wasted, respectively [4]. Undernutrition has been widely reported from urban and agrarian areas of Ethiopia [5–8], and from a limited number of studies of pastoral communities [8–10]. National-level data showed that 44 and 29 % of the children were stunted and underweight respectively [11], causing serious socio-economic burdens in Ethiopia, and being responsible for 44 % of health costs, 28 % of child mortality, 16 % of all repetitions in primary school [12].

Multifaceted factors are responsible for malnutrition, with the key underlying causes being inadequate dietary intake and other health problems, which are further rooted in socio-economic and environmental conditions. Among the socio-economic factors, poverty, food insecurity, rising cost of living, lack of access to health services and safe water supply, poor hygiene and sanitation, low income (wealth index), polygamy and large family size were the most frequently reported predictors [4, 13, 14]. Child and mother related risk factors, including age of child, male gender, low birth size, low weight of mother, low education level of mother, multiple births, high child parity, and short birth interval were also reported [4]. Other factors such as population growth, living in rural areas, unfavorable climatic conditions, drought events, lack of vaccination and diarrhea episodes have been also documented as important risk factors [4, 14].

Child growth, in terms of weight and height, has been widely recognized as an important indicator of nutritional status and health condition. The most commonly used nutritional status indicators are stunting, wasting and underweight. Stunting indicates chronic undernutrition that resulted from long-term poor diets or recurrent infections; wasting is a symptom of acute malnutrition due to insufficient dietary intake or high incidence of infections.; underweight is a composite of wasting and stunting, thus indicating acute weight loss, stunting or both conditions [15]. Accordingly, prevalence of stunting is estimated as the proportion of children with height for-age Z-score (HAZ) below − 2 standard deviations (SD). Underweight and wasting are also estimated as proportion of children with weight-for-age Z-score (WAZ) below − 2 SD and with weight-for-height Z-score (WHZ) below − 2 SD, respectively.

In Ethiopia, pastoral children were thought to have better access to foods of animal origin, which are rich in energy, proteins, and essential micronutrients. As a result, their nutritional status has been thought to be better than children in agrarian communities [11]. Pastoralism is defined as extensive livestock production on rangelands, with natural resource management and some crop growing, and in which livestock contributes to over 50 % of the household income and consumption [16]. Pastoralists use a range of key strategies (e.g. mobility, diversity, flexibility, resource conservation and strong social organization) in order to cope with environmental challenges and resources scarcities. However, several of those strategies, especially mobility, which is much reduced, have become much less effective, due to expansion of farming, establishment of administrative boundaries, enforcement of local rules, and the development of stationary infrastructures and services. These conditions have been leading pastoralists to become sedentary and to engage in crop cultivation, resulting in a phenomenon termed agro-pastoralism [17, 18]. A growing trend towards opportunistic crop cultivation, and reliance on non-pastoral income generating activities has been observed in Borana, as a means of poverty alleviation and in response to increased climate variability [18, 19]. Combined effects of increased farming, and climate variability coupled with trading milk for food grains may speed the rate of dietary transition towards a cereal-dominated diet. Consequently, children tend to consume food items that are not only low in protein, calcium and other critical micronutrients, but which also contain compounds with anti-nutrient property that reduces the bioavailability of micronutrients from consumed food items. Other studies have also demonstrated the negative impacts of reduced dairy consumption on the nutritional status of children in settled communities compared pastoralists in Kenya [20] and Nigeria [21]. Thus, investigating the extent of undernutrition among children in a pastoral setting where only limited information on nutritional status is available is vitally important for planning required nutrition intervention programs. An in-depth study of the magnitude of the problem and its determinants is necessary for developing directions for policy interventions targeting malnutrition. Accordingly, in this study we assessed the nutritional status and associated factors among infant and young children in the Borana pastoral system of southern Ethiopia.

## Methods

### Study design and sampling procedure

A community-based cross-sectional study was conducted in Borana, southern Ethiopia between August and October 2015. The area is characterized by an arid and semi-arid environment, and a pastoral/agro-pastoral production system. We applied a multistage cluster sampling method to recruit the study participants from the area. Six out of the 18 pastoral associations (PAs, the lowest administrative units), were randomly selected from Yabelo district. The district is located at the center of Borana zone, and was considered suitable for easy access to all randomly selected PAs and villages. Subsequently, about half of the villages (each with 10–20 households) were randomly sampled from each selected PAs. All eligible households in a village, who had at least one child within the age range of 6–59 months, were selected by cluster sampling. Finally, every mother and her one or more children from a selected village were enrolled for the study. Exclusion criteria were children with physical disability or abnormalities, mental impairment, edematous conditions, or evidence of chronic disease (e.g. tuberculosis), and those with signs of fever, vomiting, diarrhea and cough.

Sample size was estimated to be 561 children using presumed population proportion of minimum meal frequency (42 %) based on a report of the Ethiopian demographic and health survey [11], 95 % confidence level, marginal error of 5 % and design effect (1.5 times). Subsequently, we were able to sample a total of 538 children with complete information, while 13 children were excluded from the final dataset: Eight of them had incomplete data, three were with physical abnormalities and two were ill (showing diarrhea, fever and respiratory symptoms).

### Dietary data collection

Data on dietary intake, including dietary diversity and amount of consumed food items, dairy consumption (amount and frequency) and meal frequency were collected using 24 h dietary recalls. All food items consumed by children during the previous 24 h were listed and qualitatively described by mothers or primary caregivers. The food items were categorized according to the seven food groups model of the World Health Organization (WHO) guidelines [22]. Recipes and ingredients used to prepare the food items, dairy products and semisolid food preparations were also recorded. Accordingly, a child was considered to have received a minimum dietary diversity if she or he had consumed at least four of the seven food groups during the 24 h preceding the survey. Similarly, minimum meal frequency was considered to have been met if a child received a minimum of three meals with 1–2 snacks per day.

A questionnaire was used to collect data on socio-economic and demographic variables (family size, number of children, livestock ownership and species diversity, crop cultivation, off-farm income sources, possession of radio and mobile phone, distance from town and from public services). Similarly, maternal characteristics (age, number of children ever born, use of extra food during pregnancy or lactation, education, autonomy in decision-making), and child characteristics (age, sex, birth order, place of delivery, size at birth) were also recorded. Data on hygienic and sanitary conditions (access to sanitary facilities, hand washings and cleanings of utensils), water sources, water treatment practices, health related information (visit by health workers, vaccinations, vitamin supplement, and distance to health centers and history of illness during the three weeks prior to the visit) were also collected. We also collected information on child feeding practices, child health care, the frequency and duration of breastfeeding and intake of animal source foods.

### Anthropometric measurements

Weight and height measurements of children wearing light clothes and no shoes were recorded according to WHO guidelines. Weights were taken at standing or hanging positions using Seca scales (Seca GmbH, Hamburg Germany) with 0.1 kg resolution. Heights (standing) or lengths (lying for children below 24 months) in centimeters were measured with a measuring board graduated by 0.1 cm. The age of a child (in months) was obtained either from a birth certificate or the child’s vaccination card, with mother’s recall used for those without records. The recall was assisted by referring to the *Borana Gada* time calendar, local events, seasons, and months to determine the birthdates.

The weight and height measurements combined with age and sex of children were converted to anthropometric Z-scores of weight-for-age, height-for-age and weight-for-height according to WHO standards using Emergency Nutrition Assessment software [23]. A child with Z-scores of lower than − 2 standard deviations (SD) was regarded as malnourished i.e. underweight, or suffering from stunting or wasting [15]. These indices were regarded as proxy measures of nutritional status, and analyzed against selected independent variables. Collection of dietary data, socioeconomic variables and anthropometry data was conducted by three clinical nurses with experiences in nutritional data collections. One of the nurses, who had more experiences with anthropometric measurements than others, was regarded as a skilled enumerator. In order to minimize the measurement errors, anthropometry data of ten children were measured by the skilled nurse, and then repeated by the other two nurses. These ten groups of measurements were analyzed to evaluate the accuracy and precision of the two other nurses, using Emergency Nutrition Assessment (ENA) software [23]. The data used for this evaluation were excluded from the final dataset. Feedback of the comparative evaluation was used to improve the two less experience nurses with measuring weight and height of the study subjects.

### Data analysis

Data entry, coding and checking for errors were done using Microsoft Excel spreadsheet, and imported to Stata version 14.2 (Stata Corp. College Station, USA) for all statistical analyses. Dietary intake indicators (dietary diversity score, meal frequency, milk frequency) and socio-economic variables were descriptively summarized as percentage or mean. Most of the independent variables were categorized as binary (dummy), whereas others were regarded as quantitative variables (e.g. age of children in months, dietary diversity score, milk and meal frequency, and distance traveled in hours). A total of 40 independent variables related to socio-economic factors, dietary intake indicators, maternal and child characteristic, health and sanitary conditions were selected as potential determinants of nutritional status of the study children. The nutritional status (outcome variable) was categorizing as either malnourished (status = 1 for underweight, stunting or wasting) if a child had Z-score value below − 2 SD or regarded as normal (status = 0) when Z-score value was ≥–2SD.

The proportion of malnourished children (underweight, stunting or wasting) in study population was ≥ 10 %. In such cases, as the odds ratio tends to provide an overestimate, the strength of association of risk factors with the nutritional status of the study children was assessed using prevalence ratio (PR). Thus, we applied multivariable generalized linear model (GLM) with Poisson family, log link function and option reporting exponential coefficient. Pastoral association (PA) was entered as a cluster variable in the clustered robust option of the model. A stepwise backward selection procedure was used to retain variables by setting p ≤ 0.15 in the model. Meal frequency and sex were retained in the final model for comparison regardless of their p-value. Multi-collinearity among the independent variables was checked using variance inflation factor (VIF), and those with VIF < 9 were kept in the model.

## Results

### Socioeconomic and demographic characteristics

General characteristics of households, and of study children and their dietary data are summarized in Table 1. All households were dependent on livestock production in addition to engagement in crop cultivation (88 %) and non-pastoral income activities (25 %). Study households kept mostly cattle (62 %), with the remaining household relying mainly on camel production. Households comprised seven individuals on average, and 10 % of them were polygamous families. A large proportion of the respondents had a toilet (62 %) and at least one mobile phone (66 %) and one-third also owned one radio (35 %) per family. Few households (16.2 %) had access to developed water sources, with the majority (84 %) using unprotected sources such as ponds, deep wells or springs.

**Table 1:** General characteristics of study households (n = 406), children (n = 538) and dietary data in Borana, southern Ethiopia, August – October 2015

Variables	Unit / level	Mean ± sd / Percent (%)#
**Child characteristics**		
Age of children	in months	32.68 ± 15.25
Sex	girls	0.50 ± 0.50
WAZ	Z-score	-1.45 ± 1.03
HAZ	Z-score	-1.66 ± 1.27
WHZ	Z-score	-0.74 ± 1.03
Illness occurrence	Yes	32.0 %
Dietary diversity score	Count	2.7 ± 0.87
Milk frequency	Count	4.3 ± 1.16
Meal frequency	Count	2.3 ± 1.10
**Mother characteristics**		
Age of mothers	in years	29.39 ± 7.93
Mother education	literate	12.5 %
Polygamy	Yes	10.0 %
Decision making on resources	Yes	17.7 %
Hand washing after toilet use	Yes	71.1 %
Hand washing before food preparation	Yes	69.7 %
Received advice from health workers	Yes	83.0 %
**Household characteristics**		
Major livestock	cattle	61.7 %
Family size	number	6.59 ± 2.35
Lactating animals	in TLU*	2.49 ± 1.92
Livestock species diversity	number	2.93 ± 0.80
Off-farm income	Yes	24.7 %
Crop cultivation	Yes	88.0 %
Protected water source	Yes	16.2 %
Water treatment practices	Yes	27.1 %
Own toilet	Yes	61.9 %
Own radio	Yes	35.3 %
Own mobile phone	Yes	66.7 %
Travel time to market‡	hours	2.3 ± 3.25
Travel time to health facility‡	hours	1.1 ± 0.76
Travel time to water source‡	hours	1.4 ± 0.65

*TLU: tropical livestock unit, equivalent to 250 kg, ‡travel time is for a single trip in hours; ***#***Mean and percent (%) were indicated for quantitative and categorical (dummy) variables, respectively

Mothers or caretakers average age was 29 years, with low level of literacy (12.5 %) and limited participation in deciding on household resources (18 %). The average fertility rate was five children per mother, out of which 30 % of the children were under five years. Most of the mothers (83 %) had received extension messages from health workers about child care and feeding practices. Around 70 % of them practiced hand washing after toilet use and before preparing food or feeding their children. Some mothers (27.1 %) also reported treating drinking water (at point of use) by boiling, sand filtration or using a chemical, known locally as “Bishangari” (aluminum sulphate and calcium hypochlorite).

The study children (270 boys and 268 girls) had an average age, height and weight of 32.7 months, 86.0 cm, and 11.2 kg, respectively. The mean Z-score of weight for age, height for age, and weight for height were − 1.45, -1.66, and − 0.74, respectively. Nearly 29 % of the children were under two years of age, and 86 % of them were breastfeeding. About 4 % of the children were living with non-biological mothers, including grandmothers and relatives. Occurrences of illness (mainly diarrhea, respiratory symptoms and fever) were reported for 32 % of the children during the past three weeks before the survey.

The average milk and meal frequencies were 4.3 and 2.3 times per day, respectively. Dietary diversity score (DDS) ranged from 1 to 5 food groups with average of 2.7, so that most children (82 %) consumed less than four food groups of the WHO minimum dietary diversity. The majority of the children (68.8 %) also did not meet the WHO minimum meal frequency of at least three meals with one to two snacks per day. In contrast, a satisfactory consumption of dairy products was observed, with over 90 % of the children having dairy intake more than four times a day.

### Prevalence of malnutrition and associated factors

In order to minimize the measurement errors, anthropometry data of ten children were measured by a skilled nurse, and then repeated by the other two nurses. These ten groups of measurements were analyzed to evaluate the accuracy and precision of the two other enumerators, using Emergency Nutrition Assessment (ENA) software. Table 2 shows a summary of nutritional status of the study children based on the three conventionally used anthropometric measurements, namely height-for-age (HAZ), weight-for-age (WAZ), and weight-for-height (WHZ) Z-scores. The study showed that underweight, stunting, and wasting were prevalent in 28.3 % (95 % CI: 24.4, 32.1), 41.1 % (95 % CL: 36.7, 45.1), and 9.8 % (95 % CL: 7.3, 12.4) of the children, respectively. About 7.4 %, 14.7 and 2.2 % of the children exhibited severe underweight, stunting, and wasting, respectively. Children aged 24–59 months had higher overall prevalence of underweight (31.8 % vs. 19.7 %) and stunting (44.6 % vs. 32.8 %) compared to those 6–23 months. Wasting was 29.6 % higher in older (> 2 years) than in younger children (6–23 months). Although no significant difference was observed by sex, underweight, stunting and wasting were lower by 19 %, 12 and 24 %, respectively in girls than in boys.

**Table 2:** Prevalence (%) of underweight, stunting and wasting by sex and age groups in children (n = 538) in Borana Ethiopia, August – October 2015

Variables	Number	Underweight (WAZ)		Stunting (HAZ)		Wasting (WHZ)	
		severe (< -3SD)	overall (< -2SD)	severe (< -3SD)	overall (< -2SD)	severe (< -3SD)	overall (< -2SD)
Sex							
Boys	270	7.4	30.7	15.2	43.3	2.2	10.7
Girls	268	7.5	25.7	14.2	38.8	2.2	8.6
Age group							
6–23 months	157	3.2	19.7	11.5	31.8	3.2	7.6
24–59 months	381	9.2	31.8	15.5	44.6	1.8	10.8
Overall	538	7.4	28.3	14.7	41.1	2.2	9.8

WAZ: weight for age Z-score, HAZ: height for age Z-score, WAZ, weight for height Z-score, SD: Standard deviation

**Fig. 1:**
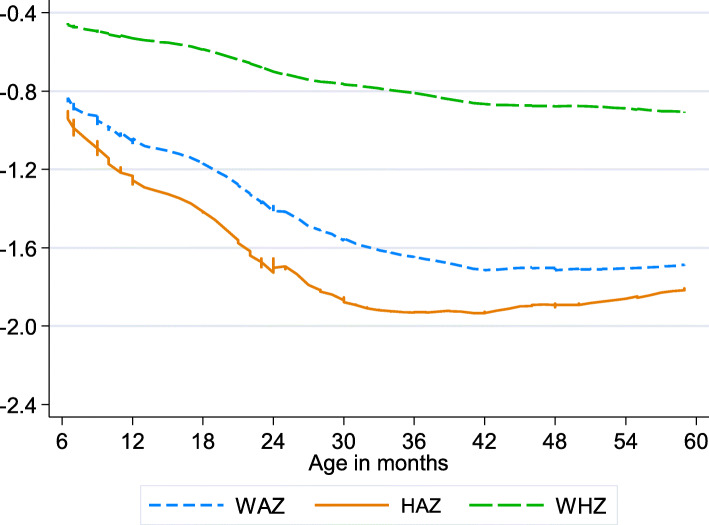
Relationship of nutritional status (anthropometric indices) with age of children in Borana Ethiopia, August – October 2015

The relationship of stunting, underweight and wasting was plotted against the age of the children (Fig. 1). Anthropometric indices initially decreased with age up to 40 months, after which WAZ and HAZ showed a slight increase.

Table 3 presents results of a multivariable analysis of potential risk factors associated with nutritional status of the study children. The results show that dietary intake, age of children, health related variables (illness, latrine ownership, proximity to health services), and distance to market were associated with malnutrition among the study children. Accordingly, children who consumed more diverse foods were at a lower risk of being underweight (PR = 0.72, 95 %CI: 0.59–0.88), stunted (PR = 0.80, 95 %CI: 0.68–0.93) and wasted (PR = 0.42, 95 %CI: 0.27–0.66) compared to those with low dietary diversity. Similarly, intake of increased milk frequency was associated with lower risk of underweight (PR = 0.86, 95 %CI: 0.76–0.97), stunting (PR = 0.83, 95 %CI: 0.75–0.91) and wasting (PR = 0.73, 95 %CI: 0.56–0.96). However, meal frequency did not have a significant effect on any of the nutritional status indicators.

**Table 3:** Factors associated with underweight, stunting and wasting among children (n = 538) in Borana, Ethiopia, August – October 2015

Variables	Unit/level	Prevalence ratio	95 % CI	Z-value	P-value
***WAZ (underweight = 28.3 %)***^***1***^					
Dietary diversity	count	0.72	0.59–0.88	-3.16	0.002
Milk frequency	count	0.86	0.76–0.97	-2.53	0.012
Meal frequency	count	1.02	0.85–1.22	0.17	0.864
Illness	yes	1.36	1.03–1.80	2.17	0.030
Travel time to health	hours	1.16	1.02–1.32	2.27	0.023
Own radio	yes	1.32	1.00–1.73	1.99	0.047
Age	month	1.02	1.01–1.03	3.62	0.000
Sex	girls	0.79	0.60–1.05	-1.76	0.078
***HAZ(stunting = 41.1 %)***^***1***^					
Dietary diversity	count	0.80	0.68–0.93	-2.88	0.004
Milk frequency	count	0.83	0.75–0.91	-3.87	0.000
Meal frequency	count	0.96	0.84–1.09	-0.62	0.535
Travel time to market	hours	1.03	1.01–1.06	2.42	0.016
Own toilet	yes	0.80	0.65–0.97	-1.98	0.049
Age	month	1.01	1.00–1.02	2.30	0.021
Sex	girls	0.90	0.74–1–09	-1.08	0.281
***WHZ (wasting = 9.8 %)***^***1***^					
Dietary diversity	count	0.42	0.27–0.66	-3.78	0.000
Milk frequency	count	0.77	0.60–0.98	-2.09	0.037
Meal frequency	count	1.19	0.97–1.44	1.69	0.092
Travel time to health	hours	1.39	1.13–1.71	3.12	0.002
Off-farm income	yes	2.88	1.67–4.96	3.81	0.000
Age	months	1.02	1.00–1.04	2.20	0.028
Sex	girls	0.75	0.52–1.08	-1.54	0.123

^1^underweight, stunting and wasting were modeled for Z-scores ≤–2SD = 1 or 0 otherwise. Meal frequency and sex were retained in the model for comparison regardless of their significance level

Age was inversely associated with nutritional status, so that underweight (PR = 1.02, 95 %CI: 1.01–1.03) stunting (PR = 1.01, 95 %CI: 1.00–1.02), and wasting (PR = 1.01, 95 %CI: 1.00–1.04) increased significantly with age. There was no significant difference between boys and girls, although girls appeared better off.

Children who lived far away from health care facilities were 1.16 times (PR = 1.16, 95 %CI: 1.00–1.73) and 1.39 times (PR = 1.39, 95 %CI: 1.00–1.73) more likely to be stunted and wasted than their counterparts. Similarly, ownership of toilet (PR = 0.67, 95 %CI: 0.46–0.98) was associated with significantly reduced risk of stunting. Children who were ill within a few weeks preceding the survey were 1.36 times more likely to be underweight (PR = 1.36, 95 %CI: 1.03–1.80) compared to those without disease symptoms. The longer the distance to a market destination the more likely the children were to become stunted (PR = 1.03, 95 %CI: 1.00–1.03). Unexpected results were also observed for children whose households had a radio or were involved in non-pastoral income sources: They had an increased risk of developing underweight (PR = 1.32, 95 %CI: 1.00–1.73) and wasting (PR = 2.88, 95 %CI: 1.67–4.96), respectively.

## Discussion

Through our study we provide empirical data on nutritional status and associated factors among children (aged 6–59 months) in the Borana community, a community that is undergoing a process of agro-pastoralism or settlement, and is increasingly engaged in crop cultivation [17, 18]. The magnitude of observed stunting (41.1 %), underweight (28.3 %), and wasting (9.8 %) are in the “very high, high and serious” categories of WHO prevalence thresholds, respectively [15]. Observed high prevalence of malnutrition can be attributed to the inadequate dietary intake of the children, most of whom (82 %) consumed below the WHO minimum dietary diversity of four food groups. Such low dietary diversity (below four food groups) have been also reported in other pastoral [24–26] and agrarian communities of Ethiopia [27–31].

Our levels of stunting and wasting are higher than the prevalence of stunting (19 %) and wasting (below 5 %) that reported by Lindtjorn et al. [32] from the same study area about two decades ago. Although comparison of two cross-sectional studies has limitations, the observed difference may indicate that the nutritional status of children in the study area is deteriorating over time, and is becoming as high as prevalence reports from mixed farming areas of the country [5–7, 33]. Observed changes in nutritional status over time (between the two studies) may be linked to the noticeable changes in Borana areas: increasing crop cultivation [18], climate variability and rangeland degradation [19], decreasing herd size per households [34], and weakening of pastoral lifestyle (e.g. mobility and flexibility) [17], in addition to human population growth. These factors in one way or another can influence household level food availability and diminish their economic access to food. In addition to low harvest rates in arid environments [18], crop cultivation has a substantial impact on livestock production due to its high competition for land, and it likely reduces intake of animal source foods. In line with this, two case studies comparing settled and mobile pastoral communities in Kenya and Nigeria have also documented the adverse effects of settlement on the nutritional status of children [20, 21].

Occurrence of stunting suggests repeated infections and/or long-term inadequate nutrient intake that often occurs in pastoral areas during dry periods and in droughts years, when dairy production and terms of trading with food grain fall [32, 35]. Thus, such very high prevalence of stunting is of great concern, as it leads to delayed motor development and impaired cognitive development that could be irreversible. Wasting, however, can be caused by acute food shortage and illness such as diarrhea (that can be improved with appropriate interventions), and may lead to child mortality. Underweight is a composite indicator that combines linear growth impediment and low weight for height, as a result of current insufficient dietary intake and illness [1]. In general, the recorded high prevalence of malnutrition calls for urgent attention as it results in poor school performance and reduced intellectual capacity of children, and leads to lower productivity as adulthood. The socio-economic burden of malnutrition in Ethiopia is evidenced by causing 44 % of the health costs, 28 % of child mortality, 16 % of all repetitions in primary school, and 67 % of the adults having suffered from childhood stunting [12].

Increased prevalence of underweight, stunting, and wasting with age of children is in agreement with previous findings [5, 7, 33, 36, 37], and could be linked to the introduction of supplemental diets of less nutrient-dense cereals. A literature review by Onyango [38] showed that stunting in African children occurs at early childhood and gets worse after two years of age, which often coincides with the introduction of less nutrient-dense supplemental diets. In addition to poor nutrient contents, cereals and tubers also contain anti-nutrient factors (e.g. phytate) which interfere with the absorption of essential micronutrients from consumed food items. Moreover, in higher ages children start to interact with the environment, and consume contaminated food and water that increase the risk of exposure to infectious agents, resulting in diarrhea episodes [4, 13].

Contrasting results can be found in the literature regarding differences between boys and girls. In several studies boys were found to suffer more from undernourishment compared to girls [5, 7, 39, 40], while others reported no difference [36, 37, 41] or found girls to be at a higher risk of malnourishment [10]. In pastoral communities such as Borana, care for children likely disfavors girls, and greater nutritional investment in girls than boys is unlikely. It is not clear whether mothers or biological differences compensate for such socio-cultural disparities by favoring girls. Possible reasons are differences in nutritional requirements as well as efficiency of nutrient conversions between girls and boys of the same age.

Dietary diversity and milk frequency emerged as major predictors of the nutritional status of study children, having significant association with lower risk of stunting, underweight, and wasting. The observed protective effects of increased dietary diversity on nutritional status of the study children confirm earlier studies’ findings [7, 42–44]. According to Motbainor et al.[7], low dietary diversity was significantly associated with higher prevalence of stunting. In another study, Steyn and colleagues [42] have demonstrated dietary diversity score to be a good estimate of nutritional adequacy and nutritional status, in that children who had low DDS were at higher risk of undernutrition. These findings imply that increasing dietary diversity results in improvement of the dietary quality of consumed food items (e.g. animal source foods) and intake of essential micronutrients that have roles in normal growth and immune system [37, 41].

Significant association of dairy intake with improved anthropometric indices is consistent with other studies in which milk consumption was associated with reduced prevalence of malnutrition and health problems, in addition to improving cognitive functions and school performances [45]. Other studies also found milk consumption to be associated with improved nutritional status of children, and to reduce the prevalence of morbidity and mortality [24, 46]. In case-control studies among school children in Iran [47] and Vietnam [45], milk consumption was significantly associated with higher anthropometric measurements of children in the intervention groups. Milk is regarded as an ultimate food that provides energy, protein, and several micronutrients and bioactive peptides with growth-promoting abilities, thus vitally enhancing the health and growth of children [48, 49]. In our study, we did not observe significant association between nutritional status and number of meals consumed per day. This could be explained by the limitation of frequency based indicator, if, for example, the same food group is frequently consumed. Cereals preparations, and to some extent beans, have been found to be the most commonly consumed foods in Borana area [26]. Hence, meal frequency does not necessarily correlate either with the extent of dietary diversity or with nutritional quality.

Other notable findings of our study were the association of nutritional status with health-related factors such as physical access to health services, availability of family toilet, and occurrence of illness during three weeks preceding the survey. Households who reside nearby health institutions might have better health information, and a higher tendency to visit health institutions and get health services compared to those living far away. It has been well documented that disease control and prevention activities through sanitation, promotion of breastfeeding, vaccination, and treatments vitally improve the health status of children, thereby contributing to normal growth and development [4, 50]. Another study in Ethiopia also documented improvement of nutritional status following immunization [51].

Observed effects of latrine ownership on reduced occurrence of stunting point to the role of improved sanitary and hygiene practices on reducing illness and malnutrition. A study in India reported lower risk of underweight and stunting among children whose households use toilets [5]. In another study, Babatunde and Qaim [52] also observed a significant association of toilet use with underweight and wasting among children in Nigeria. Toilet use indicates better sanitary conditions that reduce the risk of exposure to infections and consequent impacts on nutritional status of children. Association of illness occurrence with higher risk of underweight can be expected, as underweight reflects current poor dietary intake and illnesses - typically diarrhea and respiratory infections [1]. Bloss et al. [53] also reported that children having diarrhea, upper respiratory infections or other illnesses in the past few weeks were three folds more likely to be underweight than other groups in Kenya. Another study [5] in Ethiopia also found a significant contribution of morbidity to the increased prevalence of wasting. Reviewed studies further demonstrated illness episodes to be the most frequently reported determinants of underweight and wasting [4, 13]. In general, infections can reduce the appetite of children (reduce intake), interfering with absorption, and result in nutrient and fluid losses, thus leading to temporary weight losses.

The adverse effects of remoteness of market on nutritional status of children (increased risk of stunting) can be explained by the large share of the food supply in pastoral areas that originates from markets, affecting dietary intake. A study from Mali [54] reported positive effects of close-proximity to markets on the dietary intake of children, subsequently leading to improvement in anthropometric measures. Stifel and Minten [55] also found higher consumption expenditures and more diverse diets among households living nearer to markets in Ethiopia. Besides reducing transition cost, proximity to marketplaces may increase opportunities for household members to be engaged in non-pastoral income generating activities, or provide better access to information that may improve their nutrition knowledge. It’s worth noting that physical access to market alone may not ensure availability and access to foods, as dietary intake is mostly rooted in the socioeconomic status of households.

Surprisingly, we observed the associations of non-pastoral income sources and possession of radio with increased risk of underweight and wasting, respectively. A positive nutrition impact of off-farm income sources has been reported for farming households from Nigeria via increased household income that enables better access to food [52]. A negative effect of off-farm activities was also observed and linked to its competition for family labor and negative impact on farming activities in Mexico [56]. Our observation could be explained by the dearth of off-farm income opportunities in a pastoral area, which are mainly practiced by resource-poor households in order to stabilize their income and reduce distress selling of livestock. Those households mainly engaged in marginal off-farm income sources (e.g. petty trade, casual labor, and selling bush products), the earnings of which cannot bring about a significant effect on the nutritional status of children.

In general, malnutrition has already posed serious socio-economic burdens in Ethiopia, by incurring high health costs, child mortality, and adverse effects on academic performances [3]. The high prevalence of intestinal parasites (47–49 %) among under-five children in Ethiopia [57, 58], may contribute further to malnutrition, and need to be taken into account in a nutritional intervention program. The challenge of childhood undernutrition has already been recognized by the government of Ethiopia, so that a National Nutrition Program has been initiated to improve child nutrition through proven nutrition interventions and a national plan to end malnutrition by 2030. Thus, our findings contribute data for pastoral areas that can be used for an intended nutrition interventions under the National Nutrition Program.

## Limitations

Given the cross-sectional design of the study, it was not possible to investigate neither the temporal nor the causal relationship of dietary intake with nutritional status of children. In pastoral areas, consumption varies considerably with seasonal food availability, and we carried out this study during the minor wet season when food availability and diversity is considered to be average, and thus attempting to minimize the effects of seasonality. It would have been preferable if study subjects and units were randomly sampled from more than one district in order to extrapolate our findings beyond our study population. In addition, intestinal parasites that prevail among rural children in Ethiopia were not covered in this study. They, could have a potential role in malnutrition, and need to be taken into considerations when interpreting the effects of dietary factors on the nutritional status of the study subjects.

## Conclusions

This study is among the few from pastoral areas, and calls for attention by policymakers to address the challenges of malnutrition, specifically the observed very high prevalence of stunting, underweight and wasting. In particular, the high prevalence of stunting among pastoral children is of great concern, indicating long-term undernourishment, and calls for urgent intervention measures. Dietary diversity and dairy intake emerged as major predictors of the nutritional status of study children, thus underpinning the significance of improving mothers’ knowledge on feeding practices. Further education of mothers on good feeding and hygienic practices, particularly at weaning age may substantially alleviate the observed increased risk of malnutrition with ages, and occurrence of health problems (such as diarrhea). Association of nutritional status with access to health institutions, illness, and availability of latrine, points to the simultaneous need for improved health care and sanitation facilities in pastoral households. The Ethiopian health extension services, (encompassing various activities, including promoting toilet construction, proper hand washing, improved hygienic practices etc.) have initiated efforts to improve public health, sanitary and hygienic conditions, and child care practices in rural areas. Strengthening the existing health extension services (in terms of logistics and capacity building) would enhance the control and prevention of diseases, and ultimately contribute to effective micronutrient absorption and utilization.

## Data Availability

The datasets used and analyzed during the current study are available from the corresponding author on reasonable request.

## Abbreviations

DDS: Dietary diversity score
PA: Pastoral associations
PR: Prevalence Ratio
HA: Height-for-age Z-score
WAZ: Weight-for-age Z-score
WHZ: Weight-for-height Z-score
VIF: Variance inflation factor
WHO: World Health Organization
IMMANA: Innovative Methods and Metrics for Agriculture and Nutrition Actions

## References

[1] UNICEF-WHO-WB. The *UNICEF-WHO- WB. Joint Child Malnutrition Estimates: Levels and Trends in Child Malnutrition*.; 2012. Accessed 2019 December 04 from: http://www.who.int/nutgrowthdb/estimates2011/en/.

[2] Blössner M, de Onis M, Malnutrition (2005). Quantifying the health impact at national and local levels. WHO Environmental Burden of Disease Series no 12.

[3] Black RE, Victora CG, Walker SP (2013). Maternal and child undernutrition and overweight in low-income and middle-income countries. Lancet.

[4] Akombi BJ, Agho KE, Hall JJ, Wali N, Renzaho AMN, Merom D (2017). Stunting, wasting and underweight in Sub-Saharan Africa: A systematic review. Int J Environ Res Public Health.

[5] Zewdie T, Abebaw D (2013). Determinants of Child Malnutrition: Empirical Evidence from Kombolcha District of Eastern Hararghe. Ethiopia Q J Int Agric.

[6] Mulugeta A, Hagos F, Kruseman G (2010). Factors Contributing to Child Malnutrition in Tigray, Northern Ethiopia. East Afr Med J.

[7] Motbainor A, Worku A, Kumie A (2015). Stunting is Associated with Food Diversity while Wasting with Food Insecurity among Underfive Children in East and West Gojjam Zones of Amhara Region, Ethiopia. PLoS One.

[8] Fentaw R, Bogale A, Abebaw D (2013). Prevalence of child malnutrition in agro-pastoral households in Afar Regional State of Ethiopia. Nutr Res Pract.

[9] Gebre A, Reddy PS, Mulugeta A, Sedik Y, Kahssay M (2019). Prevalence of Malnutrition and Associated Factors among Under-Five Children in Pastoral Communities of Afar Regional State, Northeast Ethiopia : A Community-Based Cross-Sectional Study. J Nutr Metab.

[10] Maalin A, Birhanu D, Melaku S, Tolossa D, Mohammed Y, Gebremicheal K (2016). Magnitude and factors associated with malnutrition in children 6–59 months of age in Shinille Woreda, Ethiopian Somali regional state : a cross-sectional study. BMC Nutr.

[11] DHS. Ethiopian Demographic and Health Survey 2011. Central Statistical Agency, Addis Ababa:. 2011.Accessed 2015 January 09 from: http://www.measuredhs.com.

[12] WFP. The Cost of Hunger in Ethiopia. The Social and Economic Impact of Child Undernourishment in Ethiopia Summary Report. 2013. Accessed 2017 May 12 from: https://www.wfp.org/publications/cost-hunger-ethiopia.

[13] Kalu RE, Etim KD (2018). Factors associated with malnutrition among under- five children in developing countries: A review. Glob J Pure Appl Sci.

[14] Ghosh S (2020). Factors Responsible for Childhood Malnutrition: A Review of The Literature. Curr Res Nutr Food Sci.

[15] WHO. *Nutrition Landscape Information System (NLIS): Country Profile Indicators*: *interpretation guide.* Geneva,WHO. 2010. Accessed 2018 June 07 from: https://apps.who.int/iris/handle/10665/44397.

[16] FAO. *Pastoralism in Africa’s Drylands: Reducing Risks, Addressing Vulnerability and Enhancing Resilience. FAO Rome*,. 2018. Accessed 2020 December 18 from: http://www.fao.org/emergencies/resources/documents/resources-detail/en/c/1160694/.

[17] Desta S, Coppock DL (2004). Pastoralism Under Pressure: Tracking System Change in Southern Ethiopia. Hum Ecol.

[18] Tache B, Oba G (2010). Is Poverty Driving Borana Herders in Southern Ethiopia to Crop Cultivation ?. Hum Ecol.

[19] Megersa B, Markemann A, Angassa A, Valle Zárate A (2014). The role of livestock diversification in ensuring household food security under a changing climate in Borana, Ethiopia. Food Secur.

[20] Fratkin E, Roth EA, Nathan MA (2004). Pastoral sedentarization and its effects on children’s diet, health, and growth among Rendille of Northern Kenya. Hum Ecol.

[21] Ekpo UF, Omotayo AM, Dipeolu MA (2008). Prevalence of malnutrition among settled pastoral Fulani children in Southwest Nigeria. BMC Res Notes.

[22] WHO/UNICEF/IFPRI/UCDavis/FANTA/AED/USAID (2010). Indicators for assessing infant and young child feeding practices. Part II: Measurement.

[23] ENA. Emergency Nutrition Assessment for Standardized Monitoring and Assessment of Relief and Transitions (ENA for SMART) Software User Manual. 2012; Accessed 2015 March 12 from: https://smartmethodology.org/wp-content/uploads/2014/11/ENA-Manual.pdf.

[24] Iannotti L, Lesorogol C (2014). Animal milk sustains micronutrient nutrition and child anthropometry among pastoralists in Samburu, Kenya. Am J Phys Anthropol.

[25] Mengistu G, Moges T, Samuel A, Baye K (2017). Energy and nutrient intake of infants and young children in pastoralist communities of Ethiopia. Nutrition.

[26] Villa KM, Barrett CB, Just DR (2011). Whose fast and whose feast? Intra-household asymmetries in dietary diversity response among East African pastoralists. Am J Agric Econ.

[27] Aemro M, Mesele M, Birhanu Z, Atenafu A (2013). Dietary Diversity and Meal Frequency Practices among Infant and Young Children Aged 6–23 Months in Ethiopia: A Secondary Data Analysis of Ethiopian Demographic and Health Survey 2011. J Nutr Metab.

[28] Beyene M, Worku AG, Wassie MM (2015). Dietary diversity, meal frequency and associated factors among infant and young children in Northwest Ethiopia: A cross-sectional study. BMC Public Health.

[29] Dangura D, Gebremedhin S (2017). Dietary diversity and associated factors among children 6–23 months of age in Gorche district, Southern Ethiopia: Cross-sectional study. BMC Pediatr.

[30] Solomon D, Aderaw Z, Tegegne TK (2017). Minimum dietary diversity and associated factors among children aged 6–23 months in Addis Ababa, Ethiopia. Int J Equity Health.

[31] Wondafrash M, Huybregts L, Lachat C, Bouckaert KP, Kolsteren P. Dietary diversity predicts dietary quality regardless of season in 6–12-month-old infants in south-west Ethiopia Dietary diversity score and micronutrient density. *Public Health Nutr*. 2016;(14):1–10. 10.1017/S1368980016000525 PMC10270903 27041122

[32] Lindtjørn B, Alemu T, Bjorvatn B (1993). Dietary pattern and state of nutrition among children in drought-prone areas of southern Ethiopia. Ann Trop Paediatr.

[33] Yisak H, Gobena T, Mesfin F (2015). Prevalence and risk factors for under nutrition among children under five at Haramaya district, Eastern Ethiopia. BMC Pedatrics.

[34] Desta S, Coppock DL (2002). Cattle population dynamics in the southern Ethiopian rangelands, 1980–97. J Range Manag.

[35] Sadler K, Kerven C, Calo M, Manske M, Catley A (2010). The fat and the lean: review of production and use of milk by pastoralists. Pastoralism.

[36] Ayana AB, Hailemariam TW, Melke AS (2015). Determinants of acute malnutrition among children aged 6–59 months in Public Hospitals, Oromia region, West Ethiopia : a case – control study. BMC Nutr.

[37] Rose ES, Blevins M, González-calvo L (2015). Determinants of undernutrition among children aged 6 to 59 months in rural Zambézia Province, Mozambique : results of two population-based serial cross-sectional surveys. BMC Nutr.

[38] Onyango AW (2003). Dietary diversity, child nutrition and health in contemporary African communities. Comp Biochem Physiol - A Mol Integr Physiol.

[39] Mukabutera A, Thomson DR, Hedt-Gauthier BL, Basinga P, Nyirazinyoye L, Murray M (2016). Risk factors associated with underweight status in children under five: an analysis of the 2010 Rwanda Demographic Health Survey (RDHS). BMC Nutr.

[40] Aguayo VM, Nair R, Badgaiyan N, Krishna V (2016). Determinants of stunting and poor linear growth in children under 2 years of age in India: An in-depth analysis of Maharashtra’s comprehensive nutrition survey. Matern Child Nutr.

[41] Gibson RS, Ferguson EL (2008). An Interactive 24-Hour Recall for Assessing the Adequacy of Iron and Zinc Intakes in Developing Countries.

[42] Steyn NP, Nel JH, Nantel G, Kennedy G, Labadarios D (2006). Food variety and dietary diversity scores in children: Are they good indicators of dietary adequacy?. Public Health Nutr.

[43] Bruening S, Gilbride JA, Passannante SM (1999). Dietary intake and health outcomes among young children attending two urban day care centers. J Am Diet Assoc.

[44] Arimond M, Ruel MT (2018). Dietary Diversity Is Associated with Child Nutritional Status: Evidence from 11 Demographic and Health Surveys. J Nutr.

[45] Lien DTK, Nhung BT, Khan NC (2009). Impact of milk consumption on performance and health of primary school children in rural Vietnam. Asia Pac J Clin Nutr.

[46] Dror DK, Allen LH, Thi D (2011). The importance of milk and other animal-source foods for children in low-income countries. Food Nutr Bull.

[47] Rahmani K (2011). Effects of daily milk supplementation on improving the physical and mental function as well as school performance among children: Results from a school feeding program. J Res Med Sci.

[48] Hoppe C, Mølgaard C, Michaelsen KF (2006). Cow’s milk and linear growth in industrialized and developing countries. Annu Rev Nutr.

[49] Raikos V, Dassios T (2014). Health-promoting properties of bioactive peptides derived from milk proteins in infant food: A review. Dairy Sci Technol.

[50] Stephensen CB (1999). Burden of Infection on Growth Failure. J Nutr.

[51] Sinha RK (2018). Determinant of Stunting, Wasting, and Underweight in Five High-Buden Pockets of Four Indian State. Indian J Community Med.

[52] Babatunde RO, Qaim M (2010). Impact of off-farm income on food security and nutrition in Nigeria. Food Policy.

[53] Bloss E, Wainaina F, Bailey RC (2004). Prevalence and predictors of underweight, stunting, and wasting among children aged 5 and under in Western Kenya. J Trop Pediatr.

[54] Koppmair S, Kassie M, Qaim M (2017). Farm production, market access and dietary diversity in Malawi. Public Health Nutr.

[55] Stifel D, Minten B, Market, Access (2017). Well-being, and Nutrition: Evidence from Ethiopia. World Dev.

[56] Pfeiffer L, López-Feldman A, Taylor JE (2009). Is off-farm income reforming the farm? Evidence from Mexico. Agric Econ.

[57] Yoseph A, Beyene H (2020). The high prevalence of intestinal parasitic infections is associated with stunting among children aged 6–59 months in Boricha Woreda, Southern Ethiopia: a cross-sectional study. BMC Public Health.

[58] Osman KA, Zinsstag J, Tschopp R (2020). Nutritional status and intestinal parasites among young children from pastoralist communities of the Ethiopian Somali region. Matern Child Nutr.

